# Short-term effects of passive mobilization on the sublingual microcirculation and on the systemic circulation in patients with septic shock

**DOI:** 10.1186/s13613-017-0318-x

**Published:** 2017-09-08

**Authors:** Tuanny Teixeira Pinheiro, Flávio Geraldo Rezende de Freitas, Karla Tuanny Fiorese Coimbra, Vanessa Marques Ferreira Mendez, Heloísa Baccaro Rossetti, Paulo Vinicius Talma, Antônio Tonete Bafi, Flávia Ribeiro Machado

**Affiliations:** Anesthesiology, Pain and Intensive Care Department, Federal University of Sao Paulo, Napoleão de Barros 737, Sao Paulo, SP 04024002 Brazil

**Keywords:** Sepsis, Shock, septic, Physical therapy specialty, Exercise therapy, Microcirculation, Intensive care

## Abstract

**Background:**

Active mobilization is not possible in patients under deep sedation and unable to follow commands. In this scenario, passive therapy is an interesting alternative. However, in patients with septic shock, passive mobilization may have risks related to increased oxygen consumption. Our objective was to evaluate the impact of passive mobilization on sublingual microcirculation and systemic hemodynamics in patients with septic shock.

**Methods:**

We included patients who were older than 18 years, who presented with septic shock, and who were under sedation and mechanical ventilation. Passive exercise was applied for 20 min with 30 repetitions per minute. Systemic hemodynamic and microcirculatory variables were compared before (T0) and up to 10 min after (T1) passive exercise. *p* values <0.05 were considered significant.

**Results:**

We included 35 patients (median age [IQR 25–75%]: 68 [49.0–78.0] years; mean (±SD) Simplified Acute Physiologic Score (SAPS) 3 score: 66.7 ± 12.1; median [IQR 25–75%] Sequential Organ Failure Assessment (SOFA) score: 9 [7.0–12.0]). After passive mobilization, there was a slight but significant increase in proportion of perfused vessels (PPV) (T0 [IQR 25–75%]: 78.2 [70.9–81.9%]; T1 [IQR 25–75%]: 80.0 [75.2–85.1] %; *p* = 0.029), without any change in other microcirculatory variables. There was a reduction in heart rate (HR) (T0 (mean ± SD): 95.6 ± 22.0 bpm; T1 (mean ± SD): 93.8 ± 22.0 bpm; *p* < 0.040) and body temperature (T0 (mean ± SD): 36.9 ± 1.1 °C; T1 (mean ± SD): 36.7 ± 1.2 °C; *p* < 0.002) with no change in other systemic hemodynamic variables. There was no significant correlation between PPV variation and HR (*r* = −0.010, *p* = 0.955), cardiac index (*r* = 0.218, *p* = 0.215) or mean arterial pressure (*r* = 0.276, *p* = 0.109) variation.

**Conclusions:**

In patients with septic shock after the initial phase of hemodynamic resuscitation, passive exercise is not associated with relevant changes in sublingual microcirculation or systemic hemodynamics.

**Electronic supplementary material:**

The online version of this article (doi:10.1186/s13613-017-0318-x) contains supplementary material, which is available to authorized users.

## Background

Patients with sepsis who require intensive care usually receive interventions that promote immobilization, mainly the administration of sedative and analgesic drugs to facilitate invasive mechanical ventilation (MV) and reduce pain, agitation, and anxiety [1, 2]. Sepsis-induced metabolic and inflammatory changes can also lead to polyneuromyopathy characterized by a reduction in muscle force-generating capacity and atrophy and by alteration of bioenergetics [3] and sensitivity [4]. The combination of critical illness and bed rest may result in intensive care unit (ICU)-acquired weakness [5], which is associated with prolonged duration of MV, longer ICU length of stay, poor health-related quality of life, and reduced functional and cognitive capacity among survivors [4, 6–8].

Early mobilization in mechanically ventilated patients who are critically ill reduces the effects of ICU-acquired weakness [9–11]. This approach is feasible, appears to be safe [10, 12], and improves functional outcomes [10]. However, active mobilization is not possible in patients under deep sedation and unable to follow commands. In this scenario, passive therapy is an interesting alternative [13]. Passive mobilization can preserve joint mobility and muscle length [14], attenuate muscle atrophy [15], and reduce levels of inflammatory cytokines [16]. In sedated patients with septic shock, passive mobilization may have risks. In addition to accidental displacement of catheters, endotracheal tubes, enteral feeding tubes, and other supportive equipment [17], some studies have suggested that passive movements can result in a significant increase in oxygen consumption (VO_2_) and a decrease in venous oxygen saturation (SvO_2_) [18, 19]. Consequently, tissue hypoperfusion may ensue in patients with compromised cardiovascular status [18, 19]. Nonetheless, the impact of passive movements is debatable. Several other studies did not report significant effects on systemic hemodynamic variables [11, 13, 16, 17, 20–24].

No previous studies have explored the effects of mobilization on microcirculation. In these severely ill patients, it might be possible that passive mobilization can increase the blood flow to the working muscles and decrease splanchnic blood flow. These physiologic changes could lead to alterations in sublingual microvascular flow, density, and heterogeneity. However, as the relationship between systemic hemodynamics and microcirculation are not straightforward [25, 26], the assessment of microcirculation might also improve our understanding of the effects of passive mobilization beyond changes to systemic hemodynamics. We hypothesized that in patients with septic shock passive movements might be harmful by inducing significant changes in systemic hemodynamics. We also hypothesized that in these patients the changes induced by passive movements might lead to sublingual microcirculatory impairment. Thus, the objective of this study was to investigate the short-term effects of passive mobilization on sublingual microcirculation and on systemic hemodynamics variables in patients with septic shock and deep sedation.

## Methods

This study was carried out in two medical-surgical ICUs within the same university hospital. The local ethics committee approved the study (number: 1056/11), and the patients’ next of kin signed informed consent forms allowing their participation. This study was conducted according to the principles of the Declaration of Helsinki and the Brazilian National Council of Health Resolution 466/2012.

### Patients

We included patients if they met all the following criteria: 18 years of age or older, septic shock [27] with noradrenaline requirement between 0.1 and 1.0 mcg/kg/min, sedation with a Ramsay scale of 6 [28], mechanical ventilation, an arterial line for blood pressure monitoring, and an indwelling subclavian or jugular central venous catheter. All patients were included after the resuscitation phase of shock.

The exclusion criteria were intracranial hypertension, status epilepticus, or cardiac arrhythmia at the time of the study; acute myocardial infarction (Killip classification 4); thrombocytopenia (<30,000 cells/µL); hemoglobin <7.0 g/dL; leukocytosis (>50,000 cells/µL), peripheral arterial disease, worsening oxygenation status in the last 6 h (oxygen partial pressure to oxygen inspired fraction ratio decrease of >50), circulatory shock of multiple causes, or pregnant and moribund patients. We also excluded patients with medical conditions that prevented the use of a mobilization protocol, such as bone tumors, unconsolidated or unstable bone fracture, amputation, postoperative period for placement or removal of upper and lower limbs bone prosthesis, deep vein thrombosis or phlebitis, musculoskeletal deformity, and compartment syndrome in the upper or lower limbs. In addition, we excluded patients with mucosal injury or recent maxillofacial surgery that interfered with imaging acquisition.

### Study protocol

Patients were kept in a semi-recumbent position (30°). Passive movements of the upper limbs (flexion–extension of the wrist, elbow, and shoulder) and lower limbs (flexion–extension of the ankle, knee, and hip) were performed in a counter-clockwise direction starting from the upper right. The frequency of 30 movements per minute was assured by a digital metronome MA-1 BLBK^®^ (KORG, Tokyo, Japan) with 5 min for each limb, totaling 20 min of exercise. One of the investigators (TTP), a trained physiotherapist, applied the passive mobilization protocol. Although this protocol is a standard of care in our unit, it is not routinely applied in those severely ill patients.

We observed all patients for 20 min before passive movement to assure that there were no significant variations in sedative and vasoactive medication infusions as well as hemodynamic and respiratory parameters. Before the intervention, the vasopressor dose was titrated by the attending physician targeting a mean arterial pressure of at least 65 mmHg. The protocol was interrupted if any of the following adverse events occurred: detection of active movement, hypoxemia (pulse oximetry <88%), hemodynamic instability (heart rate (HR) <45 bpm, mean arterial pressure (MAP) <60 mmHg or >120 mmHg), and changes in the doses of vasoactive or sedation drugs.

### Variables

We recorded systemic hemodynamics and sublingual microcirculatory variables before (T0) and up to 10 min after (T1) passive movements. We assessed the sublingual microcirculation using sidestream dark field (SDF) imaging (Microscan^®^; MicroVision Medical, Amsterdam, Netherlands). To ensure image quality and adequate reproducibility, we adopted recommended techniques for video acquisition [29]. After removing saliva and oral secretions, the probe was applied over the mucosa. Special care was taken to avoid pressure artifacts, which was verified by checking ongoing flow in larger microvessels. We collected three high-quality steady images of at least 10 s in different sublingual regions using a portable computer and an analog-to-digital video converter (ADVC110, Canopus Co., San Jose, CA, USA). The image quality was checked before being stored at full size as DV-AVI files. Digital sequences were stored and we used the AVA 3.0^®^ software (MicroVision Medical, Amsterdam, Netherlands) for automated vascular analysis. The correct procedure for vascular video analysis with AVA 3.0^®^ software includes some manual analysis steps, including erasing incorrectly detected vessel segments and adding undetected vessel segments. Two investigators (TTP and ATB) analyzed the images off-line. The images were presented in a random order to prevent image pairing. Microcirculatory parameters included the microcirculatory flow index (MFI), the total vascular density (TVD), the De Backer score (DBS), the perfused vascular density (PVD), the proportion of perfused vessels (PPV), and the heterogeneity index (HI) [29]. To calculate the MFI score, the image was divided into four quadrants and the predominant type of flow was assessed in each quadrant using semiquantitative criteria and scored as continuous (continuous flow for at least 10 s = 3), sluggish (slow but continuous flow = 2), intermittent (no flow ≤ 50% of time = 1), or absent (no flow ≥ 50% of time = 0). The MFI score averaged the values of the four quadrants. The TVD was calculated as the length of vessels divided by total area of the image. After drawing three equidistant horizontal and three equidistant vertical lines on the screen, we calculated the De Backer score as the number of vessels crossing the lines divided by the total length of the lines. The PPV (%) can be calculated as follows: 100 × (total number of vessels − [no flow + intermittent flow])/total number of vessels. The PVD was calculated as the length of perfused vessels (flow scores of 2–3) divided by the total area of the image. We used the values generated by AVA software report. As microcirculation is heterogeneous, we calculated the HI defined as the difference between maximal and minimal PPV divided by the mean PPV value of all sublingual sites. All the variables were related to vessels with diameters less than 20 µm.

The cardiac index (CI) was measured by transpulmonary thermodilution (VolumeView/EV1000™ system, Edwards Lifesciences, Irvine, CA, USA), pulmonary thermodilution using the traditional bolus method (Edwards Lifesciences LLC, Irvine, CA, USA), or echocardiography (SonoAce R7 device, Samsung Medison, Seoul, Korea) in patients without invasive monitoring. We also collected data on age, gender, body mass index, severity scores such as Sequential Organ Failure Assessment (SOFA) and Simplified Acute Physiologic Score (SAPS) 3, admission diagnosis, Charlson index, comorbidities, source of infection, sedative drugs, vasoactive drugs, time on vasopressors, mechanical ventilation parameters, ICU length of stay, and ICU mortality.

### Statistical analysis

To calculate the sample size, we hypothesize a mean decrease of 0.25 and a standard deviation of the difference of 0.5 in the MFI after passive mobilization using 0.05 as the significance level of 0.05 with a power of 80%. Using a paired sample *t* test, the required sample size was 34 patients.

The data are expressed as a number (percent), the mean ± standard deviation, or the median (interquartile range), as appropriate. Continuous data were tested for normality by Shapiro–Wilk’s test. The systemic hemodynamic and microcirculatory variables were compared at T0 and T1 using a paired Wilcoxon test or paired *t* test, as appropriate. For the systemic hemodynamic variables, we defined post hoc as clinically relevant those changes greater than 10% using as reference the baseline [30]. As our primary hypothesis was that passive mobilization would potentially cause a worsening in the microcirculation we also hypothesize that this would occur mainly in patients using higher doses of noradrenaline. Thus, we analyzed our results according to the baseline noradrenaline dose. We classified as low-dose group dose those patients using less than 0.3 mcg/kg/min and as high-dose group those using ≥0.3 mcg/kg/min. We compared the microcirculatory variables at moments T0 and T1 within the groups, using *t* test or Wilcoxon test, as appropriate. We also measured the intensity of variation between T0 and T1 and compare the results of all microcirculatory variables between the groups using Mann–Whitney test or *t* test, as appropriate. The correlation between induced changes in systemic hemodynamics and microcirculatory variables was assessed using a Spearman correlation test.

We used SPSS version 22.0 for Windows (Armonk, NY: IBM Corp) for statistical analysis. The results with *p* values <0.05 were considered significant. For the sample size calculation, we used MedCalc software 14.12.0 (MedCalc Software bvba, Belgium).

## Results

**Fig. 1:**
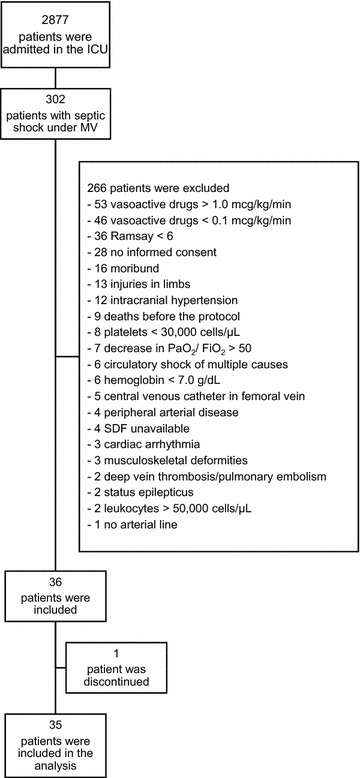
Study flowchart. ICU, intensive care unit; MV, mechanical ventilation; PaO_2_, arterial oxygen partial pressure; FiO_2_, oxygen inspired fraction; SDF, sidestream dark field

We collected data from October 2013 to May 2015. Over the study period, 2877 patients were admitted to the ICU, of whom 302 (10.5%) had septic shock and were mechanically ventilated. After the exclusion criteria were applied, 36 patients were included. However, one patient presented active movement during the protocol and was subsequently excluded (Fig. 1).

The patients included in our study were elderly (68; 49–78 years) and severely ill, as assessed by their SOFA (9; 7.0–12.0) and SAPS 3 (66.7 ± 12.1) scores. The lung was the main source of sepsis, and the mortality rate was high (60%). All patients were included after the initial phase of shock resuscitation. The patients were submitted to passive movements at approximately 39 h; 24.0–72.0 h after vasopressor infusion. The minimum duration of vasopressor infusion was 8 h. The baseline characteristics are listed in Table 1.

**Table 1:** Baseline characteristics and clinical data of the study population

Characteristics	Results (*n* = 35)
Age (years)	68 (49.0–78.0)
Gender, male	16 (45.7)
BMI (kg/m^2^)	24.6 ± 5.0
SOFA^a^	9 (7.0–12.0)
SAPS 3 score	66.7 ± 12.1
Admission diagnosis	
Surgical	19 (54.3)
Clinical	16 (45.7)
Charlson index	2.0 (1.0–6.0)
Comorbidities	
Hypertension	15 (42.9)
Diabetes mellitus	10 (28.6)
Liver failure	4 (11.4)
Source of infection	
Pulmonary	18 (51.4)
Abdominal	8 (22.9)
Blood flow	6 (17.1)
Others	3 (8.6)
Sedative drugs	
Propofol (mg/kg/h) (*n* = 30)	1.84 ± 0.83
Fentanyl (mcg/kg/h) (*n* = 28)	3.42 ± 1.41
Midazolam (mcg/kg/h) (*n* = 5)	0.08 (0.06–0.17)
Vasoactive drugs	
Norepinephrine (mcg/kg/min) (*n* = 35)	0.39 ± 0.20
Epinephrine (mcg/kg/min) (*n* = 2)	0.01 (0.01–0.01)
Dobutamine (mcg/kg/min) (*n* = 1)	2.85
Time on vasopressors pre-protocol (h)	39 (24.0–72.0)
Mechanical ventilation	
PEEP (cmH_2_O)	8.0 (6.0–10.0)
FiO_2_ (%)	40.0 (30.0–50.0)
PaO_2_/FiO_2_	239.0 ± 83.0
Cstat (mL/cmH_2_O)	33.9 ± 13.7
Tidal volume (mL/kg)^b^	7.2 ± 1.4
Driving pressure (cmH_2_O)	12.0 (9.0–16.0)
ICU LOS (days)	17.0 (12.0–25.0)
ICU mortality	21 (60.0)

The results are expressed as a number (%), the mean ± standard deviation or the median (25–75%)

BMI, body mass index; SOFA, Sequential Organ Failure Assessment; SAPS 3, Simplified Acute Physiology Score 3; PEEP, positive end-expiratory pressure; PaO_2_, arterial oxygen partial pressure; FiO_2_, oxygen inspired fraction; Cstat, static compliance; ICU, intensive care unit; LOS, length of stay

^a^SOFA was calculated on the study day, ^b^ based on predicted body weight

We obtained the cardiac index by pulmonary thermodilution (*n* = 14), transpulmonary thermodilution (*n* = 8), and transthoracic echocardiography (*n* = 12). In one patient, we were not able to record the velocity–time integral of flow through the left ventricular outflow tract. Systemic hemodynamic variables before and after passive movements are provided in Table 2. There was no significant change after exercise except for clinically non-relevant decreases in HR (T0: 95.6 ± 22.0 bpm; T1: 93.8 ± 22.0 bpm, *p* < 0.040) and body temperature (T0: 36.9 ± 1.1 °C; T1: 36.7 ± 1.2 °C, *p* < 0.002). There was no change in the lactate levels or in the central venous oxygen saturation levels.

**Table 2:** Systemic hemodynamic and ventilation variables at baseline and after exercise

Variable	Baseline	After exercise	p value
HR (bpm)	95.6 ± 22.0	93.8 ± 22.0	0.040
MAP (mmHg)	75.0 (71.0–85.0)	74.0 (69.0–84.0)	0.859
CVP (mmHg)	9.3 ± 3.6	8.7 ± 3.2	0.077
CI (L/min m^2^)	2.5 (1.7–3.6)	2.5 (1.7–3.3)	0.930
Lactate (mg/dL)	15.0 (12.0–24.0)	15.5 (11.7–23.5)	0.554
SvcO_2_ (%)	73.4 ± 8.6	72.8 ± 9.3	0.315
∆PCO_2_ (torr)	5.3 ± 1.7	5.0 ± 2.1	0.450
Body temperature, (°C)	36.9 ± 1.1	36.7 ± 1.2	0.002
PaO_2_ (torr)	91.0 (78.5–99.7)	93.3 (75.9–102.2)	0.973
PaCO_2_ (torr)	40.1 (33.9–45.7)	39.7 (32.8–48.2)	0.977
Hb (g/dL)	9.7 (8.7–11.8)	10.2 (8.6–11.9)	0.493
BE	−4.2 ± 5.4	−4.0 ± 5.6	0.297

The results are expressed as the mean ± standard deviation or the median (25–75%). Paired t test or paired Wilcoxon test

HR, heart rate; MAP, mean arterial pressure; CVP, central venous pressure; CI, cardiac index; SvcO_2_, central venous oxygen saturation; ∆PCO_2_, carbon dioxide venoarterial gradient; PaO_2_, arterial oxygen partial pressure; PaCO_2_, carbon dioxide partial pressure; Hb, hemoglobin; BE, base excess

After passive mobilization, there was a slight but significant increase in PPV (T0: 78.2 (70.9–81.9)  %; T1: 80.0 (75.2–85.1) %, *p* < 0.029), without any change in other microcirculatory variables. The microcirculatory variables are provided in Table 3. However, when we analyzed the patients according to the baseline dose of noradrenaline, only those patients using high doses at baseline had a significant increase in PPV, while those in the low-dose group did not change their PPV after the intervention (see Additional file 1: eTable 1). We tried to better understand the association between the noradrenaline dose at baseline and the changes in PPV by assessing the intensity of PPV variation (T1–T0). However, there was no significant difference between the two groups, underscoring the relevance of our previous finding of an association between noradrenaline doses and the variation in PPV (see Additional file 1: eTable 2). There was no correlation between PPV variation and the variation in systemic hemodynamics (∆HR: *r* = −0.010, *p* = 0.955, ∆CI: *r* = 0.218, *p* = 0.215, and ∆MAP: *r* = 0.276, *p* = 0.109) (see Additional file 1: eFigure 1). We did not observe adverse events during passive movements or in the recovery period.

**Table 3:** Sublingual microcirculation variables at baseline and after exercise

Variable	Baseline	After exercise	p value
MFI	2.8 (2.3–3.0)	2.8 (2.6–3.0)	0.462
TVD (mm/mm^2^)	25.6 ± 4.3	26.1 ± 4.2	0.354
DBS (n/mm)	16.0 ± 2.2	16.3 ± 1.9	0.489
PVD (mm/mm^2^)	22.4 ± 3.9	23.2 ± 3.4	0.122
PPV (%)	78.2 (70.9–81.9)	80.0 (75.2–85.1)	0.029
HI	0.15 (0.11–0.32)	0.12 (0.07–0.22)	0.149

The results are expressed as the mean ± standard deviation or the median (25–75%). Paired *t* test or paired Wilcoxon test

*MFI* microcirculatory flow index, *TVD* total vascular density, *DBS* De Backer score, *PVD* perfused vascular density, *PPV* proportion of perfused vessels, *HI* heterogeneity index

## Discussion

Our data suggest that passive mobilization of patients with septic shock after an initial phase of hemodynamic resuscitation does not compromise the microcirculation or the systemic hemodynamics. Passive mobilization was associated with an isolated and discrete improvement in the PPV. We also observed significant, although clinically non-relevant, reductions in HR and temperature.

Although passive mobilization has been widely used in the ICU over the years [31, 32], few studies have evaluated the effects of passive mobilization on hemodynamic and tissue perfusion variables. Two studies reported an increase in VO_2_ and a reduction in SvO_2_ after passive cyclic movements of the lower limbs. In our study, we were not able to find this reduction in SvcO_2_. However, one of the studies analyzed a small number of patients, which compromises the reproducibility of their results [19]. In the other, the patients were not under deep sedation; therefore, voluntary muscular activation may have occurred that could have caused the alterations. Electromyography was not performed to verify the absence of voluntary muscular contractions in these patients [18]. In contrast, in agreement with our findings, other studies showed stability or no-relevance clinical or systemic hemodynamic variables after passive exercise [11, 13, 16, 20–24].

The absence of significant changes in systemic hemodynamics can be explained by the deep sedation of our patients. Deep sedation would reduce the occurrence of adrenergic stimuli as a function of physical exercise. The passive movement applied in our study probably did not activate the exercise pressor reflex, activated by muscular ergoreceptors, which generally stimulate sympathetic autonomic activity, generating cardiovascular alterations [33]. In addition, sedation reduces the chance of voluntary muscle activity either assisting or resisting the movement performed by the physical therapist.

According to our results, microcirculatory indices were also not compromised by passive mobilization. These data are consistent with our results for systemic hemodynamics, suggesting that passive exercise does not harm patients with septic shock. We did not find previous studies that evaluated the impact of these maneuvers on microcirculation, which makes our results novel. We demonstrated that passive mobilization discretely increased the PPV, an important variable for microcirculation evaluation, since it has previously been shown as a marker of disease severity [34]. This finding was unexpected as our primary hypothesis was an impairment in the microcirculation secondary to our intervention. Although our study is underpowered to assess the potential explanations for this finding, we can provide some hypotheses. It is possible that slight changes in CI, MAP, and HR could have led to an improvement in perfusion and consequently to an improvement in PPV. However, we were unable to statistically show association between these variables. The venous return during lower extremity passive exercises usually is not sufficient to produce significant hemodynamic changes, because the patients are kept in a semi-recumbent position (30°), mobilizing less venous blood from the large splanchnic compartment. The passive leg raising hemodynamic effects also vanish after 1 min [35]. However, it is potentially possible that slight changes in the venous return, not detectable by the central venous pressure, might have contributed to a recruitment of previously closed vessels. As the intervention was made in the upper and lower limbs, a direct effect on the sublingual microcirculation is biological implausible. However, it is possible that exercise release substances, such as endorphins, have unknown effects in the microcirculation. Notwithstanding, due to our primary hypothesis, we believed that this might also be a type 1 error, and the significant difference was found only by chance, since others microcirculatory parameters did not significantly change.

Of notice, our microcirculatory variables, except the PPV, were only mildly affected, which are not a usual finding in severely ill septic shock patients with high SOFA and SAPS 3 scores and a high mortality rate. Some studies have shown that high levels of noradrenaline and the presence of hyperlactacidemia were associated with deranged microcirculatory parameters [36–38]. A possible explanation for our findings would be a dissociation between systemic hemodynamics and the microcirculation, which has already been described following resuscitation [25, 26], However, our systemic variables do not suggest this is the case. The vast majority of our patients had normal CO_2_ gradient values, SvO_2_ and lactate levels, although still using moderate doses of noradrenaline. This suggests that their mildly affected systemic variables parallel their mildly affected microcirculation. By contrary, this does not mean their survival rates would be lower. They are severely ill patients, and the determinants of sepsis survival in low- and middle-income countries are far beyond the factors analyzed in this study.

This study has some strengths. The inclusion of patients was consecutive. To the best of our knowledge, this is the first study to evaluate the impact of passive exercise on sublingual microcirculation using videomicroscopy. Microcirculation analysis is important, as studies in patients with sepsis have suggested that microcirculatory variables may remain altered despite the normalization of classic tissue perfusion variables [39]. Another relevant aspect was the technique used, as patient movement was reproduced manually by a single physical therapist, demonstrating that the protocol can be performed in places with limited resources. All four limbs were mobilized, not only the lower limbs, which is the most common practice in the ICU. In addition, all patients were severely ill and were administered vasoactive infusions and deep sedation. The use of passive mobilization in patients under minimal dose of vasopressor is already part of the current guidelines [40]. Usually, patients with higher doses would be considered by the multidisciplinary team excessively ill to be submitted to passive mobilization [41], which adds value to our findings and might stimulate its practice in this specific population. There are theoretical benefits with the use of passive mobilization [14–16]. Early mobilization in mechanically ventilated patients who are critically ill reduces the effects of ICU-acquired weakness [9–11], and this strategy includes the use of protocols of passive movements as adopted in our study.

This study also has some limitations. First, the single-center nature of our study might compromise the generalization of the results. Second, we analyzed systemic hemodynamics and microcirculation only in two periods, before and after exercise, and it is possible that changes occurred during mobilization. However, it is not possible to analyze the microcirculation during exercise. Third, our sample size does not allow a reliable analysis of the effects of different vasopressors on the microcirculation. Fourth, we found a high density and perfusion values in a very sick population and a bias in the software-assisted analysis cannot be rule out as we have consciously manually added undetected vessel segments during the analysis. However, our paired analysis minimizes the impact of this potential bias. Finally, three different methods were used to obtain CI due to ethical concerns. We used invasive monitoring only when the patient already required it for his own clinical condition. In patients without any invasive monitoring we used transthoracic echocardiography. However, we used the same technique before and after exercise in each patient, which attenuates this potential limitation as the patient was his own control and all the analysis were done using paired tests. Although cardiac output estimated by transthoracic echocardiography is not accurate and precise [42], it accurately tracks variations in CI [43] and transpulmonary thermodilution devices are comparable with the pulmonary artery catheter [44].

## Conclusions

In patients with septic shock after the initial phase of hemodynamic resuscitation, passive exercise does not seem to be associated with relevant changes in microcirculation or in the systemic hemodynamics.

## Additional file

**Additional file 1.** Microcirculation according to the noradrenaline dose and correlation between PPV variation and the variation in systemic hemodynamics.

## Abbreviations

BE: base excess
BMI: body mass index
CI: cardiac index
Cstat: static compliance
CVP: central venous pressure
DBS: De Backer score
FiO_2_: oxygen inspired fraction
Hb: hemoglobin
HI: heterogeneity index
HR: heart rate
ICU: intensive care unit
LOS: length of stay
MAP: mean arterial pressure
MFI: microcirculatory flow index
MV: mechanical ventilation
PaCO_2_: carbon dioxide partial pressure
PaO_2_: arterial oxygen partial pressure
PEEP: positive end-expiratory pressure
PVD: perfused vascular density
PPV: proportion of perfused vessels
SAPS 3: Simplified Acute Physiology Score 3
SDF: sidestream dark field
SOFA: Sequential Organ Failure Assessment
SvcO_2_: central venous oxygen saturation
SvO_2_: venous oxygen saturation
TVD: total vascular density
VO_2_: oxygen consumption
∆: delta
