# Supplementary material for: Short-term effects of passive mobilization on the sublingual microcirculation and on the systemic circulation in patients with septic shock
Source: Ann Intensive Care. 2017 Sep 8;7:95. doi: 10.1186/s13613-017-0318-x (PMC5591179; doi:10.1186/s13613-017-0318-x)
Supplement: Supplementary file 1 — Additional file 1. Microcirculation according to the noradrenaline dose and correlation between PPV variation and the variation in systemic hemodynamics. [file 13613_2017_318_MOESM1_ESM.docx]

**Additional file 1**

Short-term effects of passive mobilization in the sublingual microcirculation in patients with septic shock

Tuanny Teixeira Pinheiro, Flávio Geraldo Rezende de Freitas, Antônio Tonete Bafi, Karla Tuanny Fiorese Coimbra, Vanessa Marques Ferreira Mendez, Heloísa Baccaro Rossetti, Paulo Vinicius Talma, Flávia Ribeiro Machado

**eTable 1. Microcirculation variables according to the noradrenaline dose**

| Variable | Noradrenaline < 0,3 mcg/kg/min  (n = 13) | | | Noradrenaline ≥ 0,3 mcg/kg/min  (n= 22) | | |
| --- | --- | --- | --- | --- | --- | --- |
|  | Baseline | After exercise | p value | Baseline | After exercise | p value |
| MFI | 2.8 (2.4 – 3.0) | 2.8 (2.7 – 3.0) | 0.632 | 2.7 (2.3 – 2.9) | 2.7 (2.5 – 2.9) | 0.490 |
| TVD, mm/mm² | 26.3 ± 6.1 | 28.1 ± 5.1 | 0.139 | 25.2 ± 2.7 | 25.0 ± 3.1 | 0.668 |
| DBS_,_ n/mm | 16.1 ± 3.2 | 16.7 ± 2.4 | 0.334 | 16.0 ± 1.3 | 16.0 ± 1.4 | 0.917 |
| PVD, mm/mm² | 22.3 ± 4.9 | 23.9 ± 3.7 | 0.056 | 22.5 ± 3.3 | 22.7 ± 3.2 | 0.683 |
| PPV, % | 75.1 (68.0 - 82.8) | 78.1 (72.6 - 82.4) | 0.345 | 78.9 (72.8 - 81.9) | 80.5 (74.7 - 86.1) | **0.039** |
| HI | 0.1 (0.1 - 0.3) | 0.1 (0.0 - 0.3) | 0.753 | 0.1 (0.1 - 0.3) | 0.1 (0.0 - 0.2) | 0.123 |

MFI - microcirculatory flow index; TVD - total vascular density; DBS - De Backer score; PVD - perfused vascular density; PPV - proportion of perfused vessels; HI - heterogeneity index. The results are expressed as the mean ± standard deviation or the median (25% - 75%). T-test or Wilcoxon test. There were no significant differences in the baseline values for MFI (p = 0.497), TVD (p = 0.570), DBS (p = 0.936), PVD (p = 0.895), PPV (p = 0.306), HI (p = 0.473).

**eTable 2. Microcirculation deltas according to the noradrenaline dose**

| Variable | Noradrenaline  < 0,3 mcg/kg/min  (n = 13) | Noradrenaline  ≥ 0,3 mcg/kg/min  (n= 22) | p value |
| --- | --- | --- | --- |
| Delta MFI | 0.0 (-0.1 - 0.4) | 0.0 (-0.1 - 0.1) | 0.756 |
| Delta TVD | 1.8 ± 4.2 | -0.5 ± 2.7 | 0.106 |
| Delta DBS | 0.6 ± 2.3 | -0.4 ± 1.8 | 0.215 |
| Delta PVD | 1.5 ± 2.7 | 0.4 ± 2.8 | 0.320 |
| Delta PPV | 2.9 (-1.2 - 5.8) | 4.1 (-2.6 - 6.6) | 0.657 |
| Delta HI | -0.0 (-0.1 - 0.1) | -0.0 (-0.1 - 0.3) | 0.596 |

MFI - microcirculatory flow index; TVD - total vascular density; DBS - De Backer score; PVD - perfused vascular density; PPV - proportion of perfused vessels; HI - heterogeneity index. The results are expressed as the mean ± standard deviation or the median (25% - 75%). T-test or Mann-Whitney test


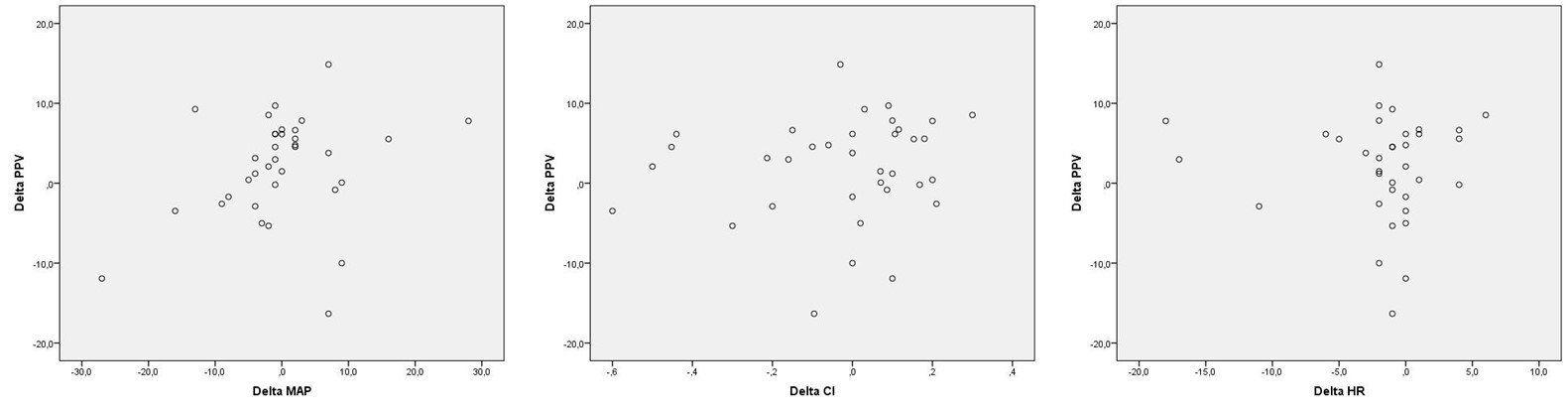


**Figure S1. Correlation between proportion of perfused vessels (PPV) variation and the variation in mean arterial pressure (MAP), cardiac index (CI) and heart rate (HR).** There is no significant correlation: ∆MAP:r = 0.276, p = 0.109, ∆CI:r = 0.218, p = 0.215 and ∆HR: r = - 0.010, p = 0.955.
